# Embodied Heuristics

**DOI:** 10.3389/fpsyg.2021.711289

**Published:** 2021-09-30

**Authors:** Gerd Gigerenzer

**Affiliations:** Max Planck Institute for Human Development, Berlin, Germany

**Keywords:** embodied heuristics, gaze heuristic, interception problems, sensorimotor abilities, bounded rationality, adaptive toolbox

## Abstract

Intelligence evolved to cope with situations of uncertainty generated by nature, predators, and the behavior of conspecifics. To this end, humans and other animals acquired special abilities, including heuristics that allow for swift action in face of scarce information. In this article, I introduce the concept of *embodied heuristics,* that is, innate or learned rules of thumb that exploit evolved sensory and motor abilities in order to facilitate superior decisions. I provide a case study of the gaze heuristic, which solves coordination problems from intercepting prey to catching a fly ball. Various species have adapted this heuristic to their specific sensorimotor abilities, such as vision, echolocation, running, and flying. Humans have enlisted it for solving tasks beyond its original purpose, a process akin to *exaptation*. The gaze heuristic also made its way into rocket technology. I propose a systematic study of embodied heuristics as a research framework for situated cognition and embodied bounded rationality.

## Brief Summary

Bounded rationality is the study of how humans and other animals rely on heuristics to achieve their goals in situations of uncertainty. It differs from axiomatic rationality, which asks whether humans conform to logical principles such as transitivity. This paper contributes to the emerging field of embodied bounded rationality, which studies how the body supports rational behavior. Specifically, I propose the concept of embodied heuristics, along with a program on how to study these. An embodied heuristic requires specific sensory and motor abilities to be executed. I provide a case study of the gaze heuristic, which solves visuomotor coordination problems when capturing or avoiding a moving target, from intercepting prey to catching a Frisbee. I show how various species adapted the heuristic to their specific sensory and motor abilities, allowing it to solve interception problems in both two dimensions (on the ground) and three dimensions (in the air or water), and for vision and echolocation. Humans rely on the heuristic for catching fly balls and other tasks beyond its original domain, a process akin to exaptation. The heuristic has been built into rocket technology. This article is of programmatic nature, outlining a novel research program for situated cognition and embodied bounded rationality.

## Introduction

Jean Piaget once said that he cannot think without a pen in hand. For him, writing *was* thinking, not the translation of thought onto paper (Gruber and Vonèche, 1977). Accordingly, his theory of cognitive development begins with the child’s sensory and motor processes, which are eventually transformed into mental life, where they become cognitive operations and structures. The general idea that cognition is closely intertwined with action was later called *embodied cognition*. This term, however, has been used for a highly diverse set of ideas, including the role of gestures, narratives, and physical proximity in behavior. An early version was *ecological psychology*, most prominently Gibson’s (1979) view that perception requires movement to detect the invariants in ambient light: “So we must perceive in order to move, but we must also move in order to perceive” (p. 223). In the field of robotics, Brooks (1991) embraced a Gibsonian-inspired architecture, where robots need no symbolic representation of their world; their sensors are connected directly to their behaviors, enabling them to “use the world as its own model” (p. 139). What unites these various approaches, which have been called the four “E’s” – *embodied*, *embedded*, *extended*, and *enactive cognition* – is their critique of theories that explain behavior on the basis of internal processes only (e.g., theory of mind or computational theories of cognition) without considering the role of the body and the environment (Wilson, 2002; Shapiro and Spaulding, 2021).

In the present article, I begin from a different perspective, the evolution of rational behavior.

One might think that rational choice theory – choice axioms and subjective expected utility maximization – has long investigated how humans and other animals make decisions. Yet most theories of rational behavior assume that humans have mental capacities for foreseeing the future that real humans can only dream of: perfect foresight of all future events, along with their consequences and probabilities (Hammerstein, 2012). These assumptions are made not because they are realistic but because they are needed to apply the convenient mathematical tools of optimization. Economist Milton Friedman (1953) famously defended these “as-if” models by arguing that their purpose is prediction, not psychological realism. Their strength lies in the beauty of abstract models, where humans are pictured as econometricians. Their downside is that everything psychological plays little if any role, except as a source of irrationality. This methodological choice has left us with an unsatisfying situation. It has promoted a flood of theories that neither describe actual behavior nor intend to do so. Furthermore, contrary to Friedman’s vision, expected utility models appear barely able to predict behavior. According to a review, “their power to predict out-of-sample is in the poor-to-nonexistent range” (D. Friedman et al., 2014). Logical axioms hence may not have been the best route to understanding rational behavior in the real world.

In this article, I start with an evolutionary view on decision-making. I introduce the concept of *embodied heuristics*, that is, rules of thumb that exploit specific sensory and motor capacities in order to facilitate high-quality decisions in an uncertain world. Instead of taking an axiomatic approach, models of heuristics take an *algorithmic* approach to represent the sequential process of decision-making in time. Following that, I present a case study of the gaze heuristic that illustrates how an embodied heuristic exploits sensory and motor abilities and how the heuristic has been adapted to the specific abilities of different species. Moreover, by a process akin to exaptation, the heuristic ended up solving new tasks created by human culture. I begin with what might have been the first decisions made by living organisms.

## The Dawn of Decision-Making

The earth is about 4.5 billion years old. Life emerged some 3.8 billion years ago and animals much later, about 1 billion years ago. It began in the form of single-celled organisms equipped with early versions of sensors and a small repertoire of actions. The best-studied single-celled organism is a bacterium called *E. coli* (named after its discoverer, the pediatrician Theodor Escherich). Its popularity is based on the observation that it does not appear to die but instead splits into two daughter bacteria, which again split, and so on (Khamsi, 2005). It can be found in the lower intestine of humans and other warm-blooded organisms. *E. coli* can perform two motions, run or tumble, that is, move in a straight line or randomly change course. It continuously switches between these actions, although tumbling is reduced when its sensors detect increasing concentrations of food (see Godfrey-Smith, 2016, for a philosopher’s account of this behavior). Here we observe the earliest form of decision-making: bacteria choosing between two actions, run or tumble, guided by chemical cues in their environment. These actions serve adaptive goals, finding food and avoiding toxins. The bacteria rely on decreasing or increasing rates of various chemicals as cues. In decision theory, a cue is a sign, or clue, of something that is not directly accessible, such as food or toxins.

Bacteria are *prokaryotes*, cells without a nucleus. Much later, *eukaryotes* arose from a merger of bacterial cells and eventually formed plants, mushrooms, and animals. Eukaryotes also formed “eyespots,” which mark the beginning of vision and allow for further cues to guide action. One of these, light, has a dual function. For some organisms such as single-celled organisms and plants, it is mainly a source of energy, supplying solar power. Although humans and other animals also sunbathe, for them light is primarily a source of information. Humans *infer* the outside world from patterns of light.

Inference is crucial, as we cannot directly see the world. Our inferences, albeit more elaborate than those of single cells, remain intelligent “bets” based on uncertain cues. The great physiologist Hermann von Helmholtz spoke of “unconscious inferences” because even humans are not aware of how they make these inferences, such as reconstructing a three-dimensional world from a two-dimensional retinal image. Unconscious inferences border on magic, given that an infinite number of states of the world are consistent with this retinal image. Through millions of years of learning, sensory and motor abilities have evolved in tandem with heuristics that help make good inferences in such situations of uncertainty – to find food and mates, to avoid toxins and predators, and to solve the basic goals of organisms.

Along with individual inferences, social behavior evolved. Consider *E. coli* again. It reacts not only to signs of edible food and dangerous toxins, but also to chemicals that signal the presence of other bacteria. This reaction opened the door to the evolution of *coordination* between organisms, that is, social behavior. An example is *quorum sensing* among bacteria living inside of squids. Bacteria produce light through a chemical reaction, but only if enough other bacteria are around to join in. They appear to follow a simple heuristic: The more of the signaling chemical one senses, the more light one produces (Godfrey-Smith, 2016, p. 19). The light produced serves its host, the squid, as camouflage. Without this light, predators from below would see the shadow of squids, which are nocturnal animals, as cast by the moonlight. In humans, social coordination takes many forms, including communication, cooperation, and competition, and has led to cultural systems such as churches, political parties, and the market.

Let us now consider a concrete example of how inferences are made based on an embodied heuristic.

## Embodied Heuristics: an Illustration

Ants, like humans, make real-estate choices, that is, decisions about where to live, which are essential to their fitness. Consider *Leptothorax albipennis*, a small ant approximately 3mm long that lives in colonies with up to 500 workers and a single queen. When their old nest is destroyed, the ant colony sends out scouts to locate a new site that is sufficiently large to house the entire colony. The ants prefer nest sites consisting of narrow cracks in rocks with flat areas. How can a scout ant estimate the irregular area of a candidate site? A series of ingenious experiments revealed that scout ants use a smart rule called “Buffon’s needle algorithm,” named after the French eighteenth-century mathematician Buffon, who discovered it millennia after the ants did (Mallon and Franks, 2000).

To determine the size of the area, the scout ant first moves for a fixed period (less than two minutes) on an irregular path that covers the area fairly evenly. While doing so, it leaves behind a trail of pheromones. After that the ant exits the area, and then returns and repeats the procedure of walking around randomly. In this second round, the ant counts how often it crosses its own pheromone trail and uses the count to estimate the area of the site: the larger the number of crossings, the smaller the area. This heuristic is amazingly accurate: For a site that is half the size of the area needed, the frequency of crossing is 1.96 times greater (Mugford et al., 2001).

In Buffon’s needle problem, the question is asked, what is the probability *p* that a needle dropped on a floor made of parallel and equally wide strips of wood will end up lying across a line between two strips? For a needle of length *l*, *p*=*2l/πt*, where *t* is the width of the strips. Buffon used the solution to calculate the number π. In the ant’s heuristic, the lines are the ant’s pheromone trail and the needles lying across lines are the ant’s crossings of its own trail. The ant is not interested in π, but in the length *t* between lines, which indicates the area.

The ant’s heuristic involves its body in several ways. First, the ant needs to move around. The heuristic would not work if the ant simply sat still and looked around. Second, the ant’s body produces a pheromone trail, and its sensory system has the ability to recognize its own trail. These biological functions are necessary for the heuristic to be executed, but not sufficient. In addition, the ant needs cognitive abilities such as counting crossings and retaining a memory of the count. Many insects can in fact measure and memorize the rate at which they encounter stimuli (Stephens and Krebs, 1986). All in all, ants have evolved an embodied heuristic to infer the area of potential nest sites.

## Axiomatic Rationality, Bounded Rationality, and Ecological Rationality

The scout ant solves an adaptive problem, finding a nest site. The bacteria *E. coli* solves its own adaptive problems, finding food and avoiding toxins. Adaptive problems relate to survival and reproduction, such as finding a safe location, food, and a sexual partner, or cooperating and competing in social groups (Tooby and Cosmides, 1992). A common characteristic of adaptive problems is the presence of uncertainty, that is, when full knowledge of all options together with their consequences and probabilities is not attainable. Theories of rational behavior, in contrast, have mostly studied artificial lotteries and well-defined games where all is known for certain, including the probabilities. These are known as situations of risk (Knight, 1921).

### Axiomatic Rationality

The best-known theory of decision-making goes by many names: axiomatic rationality, expected utility maximization, or rational choice theory. Given the many definitions of rational choice theory, *a*x*iomatic rationality* is a more precise designation. In the axiomatic approach, the term *rationality* has little to do with solving adaptive problems. Instead, it refers to a set of choice axioms, such as completeness and transitivity, and to expected utility maximization. It is also not meant to describe the process of how ants choose a new nest site or how humans make decisions. All it might offer is a model in which ants are assumed to have complete knowledge about the features of all sites in reach and choose a site that maximizes their utility. Such a model would neither help an ant to know what to do nor aid a behavioral biologist in understanding what ants do nor guide an AI engineer in building a robot ant. The theory is deliberately abstract and “as-if.”

In fact, its originators, von Neumann and Morgenstern (1944), never intended axiomatic rationality to describe what humans and other animals do or what they should do. Instead, these authors derived the necessary and sufficient conditions to represent choices on a number line, called *utility function*. These conditions are the choice axioms and are similar to the properties of real numbers (Cantor, 1954). Von Neumann and Morgenstern’s great contribution was to prove that if an individual satisfies the set of axioms, then their choices can be represented by a utility function – *nothing more*. Nowhere in the three editions of their landmark book did the founders speak of axioms as a description of how people behave or should behave.

Ten years later, the normative interpretation of choice axioms was promoted by Savage (1954), known as the father of Bayesian decision theory. Yet Savage explicitly limited the theory to *small worlds (S, C),* that is, situations in which the exhaustive and mutually exclusive set of future states *S* and their consequences *C* are known. That is why choices between lotteries have become a standard task in decision research, from behavioral economics to cognitive neuroscience. Here, all future states (the tickets), their outcomes (the prizes), and their probabilities are known (as mentioned before, these are also called *situations of risk*.) However, Savage maintained that it would be “utterly ridiculous” (p. 16) to apply utility theory beyond small worlds, that is, to well-defined situations that are intractable, such as chess, or to ill-defined situations where one cannot know all possible future states and their consequences. Savage’s example of an ill-defined situation was planning a picnic (p. 16), which is prone to unexpected events.

In a surprising turn in history, quite a few psychologists and economists disregarded Savage’s restrictions and began to assert that axiomatic rationality applied to *all* situations (Binmore, 2008). At the same time, it was known since the demonstrations of Maurice Allais and Daniel Ellsberg that people systematically violate the theory *even in small worlds*. Note that both Allais and Ellsberg criticized the rationality of rational choice theory, not the rationality of people. Nevertheless, many psychologists (mis)construed the theory to be normative and routinely blamed deviations on people, not the theory (e.g., Thaler and Sunstein, 2008; Kahneman, 2011). These deviations were attributed to *bounded rationality*, implying that humans are innately susceptible to cognitive illusions or even irrationality.

### Bounded Rationality and Ecological Rationality

But that was not what Herbert Simon, who coined the term *bounded rationality*, meant. In fact, Simon (1989, p. 377) argued in favor of studying how humans and other animals actually make decisions when the conditions for axiomatic rationality are not met, that is, under uncertainty. His revolutionary proposal required leaving the safe haven of small worlds, or situations of risk, and sailing out to study the actual process of decision-making under uncertainty. That proved too much for most neo-classical economists, who reinterpreted bounded rationality as optimization under constraints, which is also not what Simon meant. The psychologists who studied deviations from axiomatic rationality attached yet another meaning to bounded rationality, as deviations between judgment and rational choice theory that signify irrationality. While these latter two definitions contradict each other, one signifying rationality, the other irrationality, what they share is their embrace of rational choice theory as the unconditional benchmark for all behavior (Gigerenzer, 2020). This double takeover has been so successful that few people have noticed how *bounded rationality* has been decoupled from Simon’s revolutionary program.

**Figure 1:**
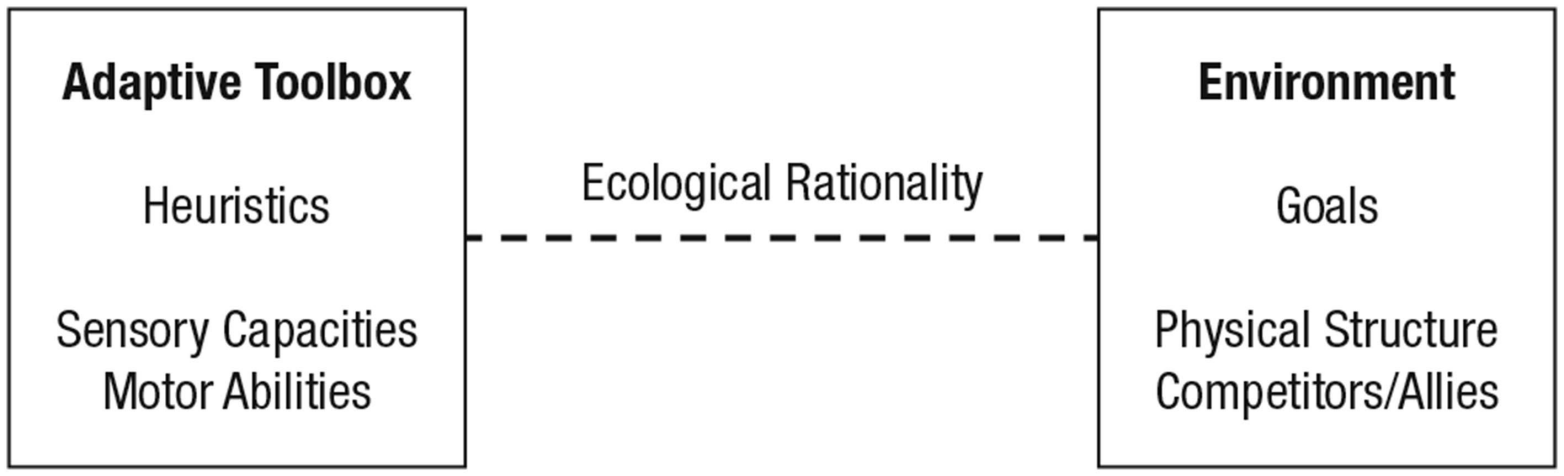
Rationality as the match between heuristics and environment. Left side: The adaptive toolbox of an individual or species, with heuristics that are embodied in sensory capacities and motor abilities. Right side: The environment, including the goals of individual or species and their physical and social structure. The ecological rationality of a heuristic is measured by the degree to which it can attain a goal.

As in axiomatic decision theory, the study of the mind’s evolved psychology, not to speak of the body, appears irrelevant for human decision-making in the present definitions of bounded rationality. To avoid confusion, my colleagues and I instead refer to *ecological rationality* in our work on extending Simon’s original program (Gigerenzer et al., 1999). Figure 1 shows the general framework. The left side represents mind and body; the right side represents the environment in which a decision needs to be made. These two sides specify the two blades of Simon’s “scissors,” an analogy he used to explain why one needs to investigate the interplay between cognition and environment to understand behavior: Looking at only one blade of a pair of scissors does not explain how it cuts so well.

The study of ecological rationality analyzes the match between the adaptive toolbox of an individual or species, and the environment. A *match* refers to the likelihood that a given heuristic achieves a given goal in a given environment. Heuristics exploit sensory capacities and motor abilities and are in this sense embodied heuristics. Together, they constitute the adaptive toolbox, which specifies the first blade of Simon’s scissors.

The second blade is the environment. It contains the goals of the organism, such as a good nest site. Note that the *environment* here refers to the world as experienced by animals or humans, as in von Uexküll’s (1957) *Umwelt*, not to an exhaustive description in terms of molecular biology or geophysics.

The study of ecological rationality addresses three questions (Gigerenzer and Gaissmaier, 2011; Todd et al., 2012). The first concerns the repertoire of tools: What are the heuristics in the adaptive toolbox of an individual, institution, or species? The second concerns the organism’s environment: What are the relevant environment structures? The third concerns the match between mind and environment: What are the environmental conditions conducive to the success of particular heuristics with respect to a goal? Together, the answers to these three questions enable us to comprehend why heuristics evolved and the conditions under which a given heuristic is likely to succeed.

What the study of ecological rationality does *not* ask is whether a behavior departs from logical systems of rationality. Strictly following logical inference can, in fact, even hinder solving adaptive problems. Consider two parties engaged in a social contract of the type “if you take the benefit, then you have to pay the costs” (Cosmides, 1989). Although the heuristic “check whether your partner took the benefit but did not pay the costs” can lead to choices that contradict an interpretation of the social contract as a logical conditional “if p then q,” it enables detecting cheaters (Gigerenzer and Hug, 1992). Similarly, a review of deviations from choice axioms and other logical rules – often interpreted as cognitive illusions – found little to no evidence that these deviations are actually associated with lesser health, wealth, happiness, or any other measurable costs (Arkes et al., 2016).

Unlike the ant’s implementation of Buffon’s needle algorithm, many models of heuristics do not make reference to specific sensory or motor abilities. An example is the investment heuristic 1/N, which solves the problem of how to invest a sum of money into *N* assets by allocating it equally. In the uncertain world of stocks, this fast-and-frugal heuristic has been shown to be able to outperform the Nobel Prize-winning mean-variance portfolio (DeMiguel et al., 2009). However, 1/N does not specify or require specific sensorimotor abilities; dividing a sum by the number of assets can be performed by a pocket calculator as well. Similarly, heuristics such as *minimax* (determine the worst outcome of each option and choose the option with the least undesirable outcome) and *tallying* (count the positive reasons for each option and choose the option with the highest number) do not specify or require any abilities apart from calculation (Gigerenzer and Gaissmaier, 2011).

I will reserve the term *embodied heuristic* for rules that require specific sensory and/or motor abilities to be executed, not for rules that merely simplify calculations. In the next section, I describe in more detail an embodied heuristic that humans share with animal species.

## The Gaze Heuristic

When faced with a ball high up in the air, experienced baseball outfielders know where to run in order to catch it. How do they solve the task? There are two visions for finding an answer. The first is to treat the question as an optimal control problem and assume close-to-omniscient players who can make complex calculations unconsciously. That is how Richard Dawkins (1989), p. 95) thinks a player catches a ball:

He behaves as if he had solved a set of differential equations in predicting the trajectory of the ball. He may neither know nor care what a differential equation is, but this does not affect his skill with the ball. At some subconscious level, something functionally equivalent to the mathematical calculations is going on.

To determine the trajectory of the ball, consciously or unconsciously, the player has to estimate the parameters in this formula:


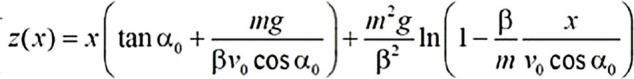
 (1)

where *z*(*x*) is the height of the ball at flight distance x, measured from the position where the ball was thrown. At *z*(*x*)=0, the ball hits the ground. To calculate *z*(*x*), the player has to estimate both the initial angle α_0_ of the ball’s direction relative to the ground and the initial speed v_0_ of the ball; know the ball’s mass *m*, the friction *β*, and that the acceleration of earth *g* is 9.81m/s^2^ (meter/s squared); and be able to calculate tangent and cosine. Even then, the formula is overly simplified in that it considers only two dimensions and ignores wind and spin. Importantly, the true challenge is not computing the equation, but *estimating* its parameters, such as the initial angle and the initial speed.

**Figure 2:**
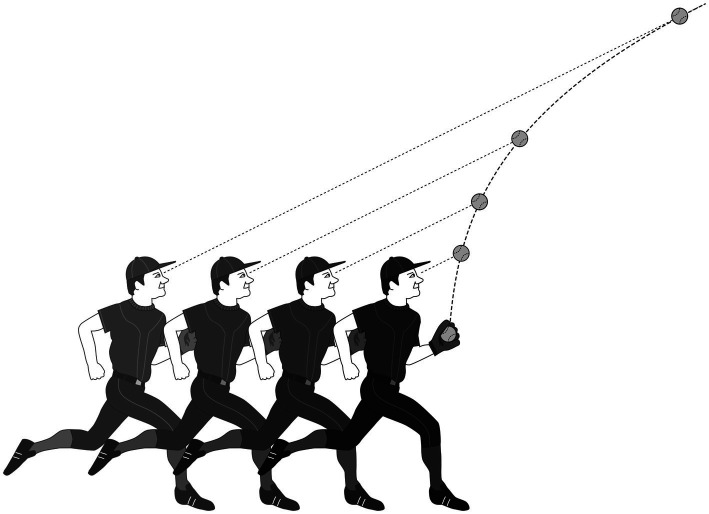
Gaze heuristic. The player adjusts the running speed so that the angle of gaze remains constant. The angle of gaze is the angle between the line from eye to ball and the ground. Shown is the player’s position relative to the ball for four points in time.

Note that Dawkins put the term “as if” into his explanation of how players solve the goal. He was well aware that players do not calculate trajectories; they only behave *as if* they did. What players actually do at the subconscious level remains a mystery in his account. Yet that mystery has been resolved by experimental studies. Experienced players catch a fly ball by using a heuristic that has absolutely nothing to do with calculating a trajectory (Figure 2).

Gaze heuristic: *Fixate your eyes on the ball, run, and adjust your speed so that the angle of gaze remains constant.*

The gaze heuristic ignores all the information necessary for computing a trajectory and attends to one variable only, the angle of gaze. In this way, it avoids any measurement errors when estimating the parameters in Equation 1. It consists of three “building blocks” – fixating, running, and adjusting – and works in situations where the ball is already high in the air. If that is not the case, the player needs to adapt the third building block:

*Fixate your eyes on the ball, run, and adjust your speed so that image of the ball rises at a constant rate.*

One can easily see the logic. If the image of the ball rises at an accelerating rate, the ball will hit the ground behind the player’s present position, meaning that the player needs to run backward. If it rises at a decreasing rate, the ball will hit the ground before the player, who then needs to run faster. If the image of the ball rises at a constant rate, the player is running at the correct speed (McBeath et al., 1995; Shaffer and McBeath, 2002).

The gaze heuristic is an embodied heuristic. It requires the ability to hold one’s gaze on an object, to run, and to adjust one’s running speed. These abilities are learned early in development. For instance, babies begin to exercise visual tracking of moving objects around 2months of age, such as tracking the objects in mobiles (Jonsson and von Hofsten, 2003). The body is part of the solution. In contrast, state-of-the-art bipedal robots cannot implement the gaze heuristic because they lack the ability to run and to securely hold their gaze on a moving object against a noisy background.

### Predicting Behavior: As-if Models vs. Embodied Heuristics

Let me now make two more general points. First, reliance on as-if models rather than process models can mislead researchers regarding the actual goal of an organism. The trajectory calculation model suggests that the player’s goal is to determine the point where the ball hits the ground (or is at a height in reach of the player) and then run to this point (Table 1). The gaze heuristic, in contrast, implies that the goal is to intercept the ball. No knowledge about the landing point is necessary; the heuristic leads the player to the ball. A heuristic is not a just an efficient means toward a given end. It can specify what exactly the player wants to achieve. Means can determine ends, not just the other way round.

**Table 1:** The trajectory calculation model and the gaze heuristic make different predictions about both behavior and cognitive processes. In addition, they imply different specifications of the player’s goal. The checkmarks show the predictions supported by experimental studies.

	Trajectory Calculation	Gaze Heuristic
Player’s goal	Compute landing point	Intercept ball
Prediction 1: Speed	*Runs full speed to landing point.*	*The angle of gaze controls* *running speed and its change.✓*
Prediction 2: Interception	*At the landing point, player waits to catch ball.*	*Intercepts ball while running.✓*
Prediction 3: Course	*Runs in a straight line.*	*Runs in a slight arc.✓*
Prediction 4: Landing point	*Knows where the ball is landing.*	*Does not know landing point.✓*

Now consider the argument by Milton Friedman that models need not be concerned with psychological realism, only with good predictions. The gaze heuristic and the study of embodied heuristics in general, however, show that psychological realism can lead to better predictions than as-if models. Because as-if models do not care about cognition, only about behavior, let us have a closer look at four predictions about behavior (Table 1).

Consider first the running speed. The trajectory model suggests that players would perform better the faster they run to the expected landing point, so that they have time for last-second adjustments. In contrast, the gaze heuristic makes a very specific prediction: that players’ speed is controlled by the angle of gaze, which determines speed and its change. If players run too fast, they will miss the ball.

Second, consider interception. According to the trajectory model, players should ideally arrive at the landing point before the ball and wait for it. The gaze heuristic, in contrast, implies that players catch the ball while running. The reason is that they adjust their running speed until they catch the ball. In both cases, the predictions following from the gaze heuristic have been supported by experimental studies (e.g., McBeath et al., 1995; Shaffer and McBeath, 2002).

Third, consider the course of running. According to the trajectory model, the player will run straight toward the landing point. In contrast, the gaze heuristic can imply in certain situations that players run a slight arc to keep the angle of gaze constant. These arcs have been demonstrated in experiments with skilled outfielders (Shaffer and McBeath, 2002).

Finally, if players consciously or unconsciously computed the landing point, as assumed by the trajectory model, they would know where the ball will land. No such knowledge is implied by the gaze heuristic. Studies show that even experienced players (just like ordinary people) have difficulties estimating the trajectory of the ball, its apex, and the landing point yet are nevertheless able to catch the ball (Shaffer and McBeath, 2005).

The general point is that the as-if trajectory model is ignorant about the process and objectives of decision-making and thus makes incorrect predictions about the resulting behavior. It treats the problem as one of calculating landing points, while the heuristic treats it as one of coordination between body and ball.

### Coordination Problems

The gaze heuristic and its relatives can resolve various coordination problems. These include interception, such as when athletes catch balls, but also avoidance of collisions, as in sailing and flying. When beginners learn to sail, they are taught a version of the gaze heuristic to infer whether another boat is on a collision course: Fixate your gaze on the other boat; if the angle of gaze remains constant, change your course quickly. When beginners learn to fly a light aircraft, they may be taught a further version of the same rule: If another plane approaches and you fear collision, look at a scratch in your windshield and observe whether the other plane moves relative to that scratch. If not, dive away immediately – otherwise, the plane might end up colliding with this scratch.

The “miracle on the Hudson River” is a famous case where reliance on the gaze heuristic saved lives. On January 15, 2009, US Airways Flight 1549 collided with a flock of Canada geese shortly after take-off, which shut down both engines. The pilots had to make a life-and-death decision: to try to reach the next airport or attempt a risky landing in the Hudson. Landing at the next airport would have been the safer option, but only if the plane could actually make it that far. As co-pilot Jeffrey Skiles explained, to determine whether the sailing plane could safely make it to the airport, they did not try to calculate the trajectory of the plane but instead relied on a version of the gaze heuristic (Rose, 2009):

It’s not so much a mathematical calculation as visual, in that when you are flying in an airplane, a point that you can’t reach will actually rise in your windshield. A point that you are going to overfly will descend in your windshield.

The point in the windshield rose, which meant the plane would have crashed before reaching the airport. The heuristic helped to make the right decision; all passengers and crew survived (Gigerenzer, 2014, pp. 27–29).

Note that the heuristic can be used both consciously and unconsciously, as illustrated by the pilots and the outfielders, respectively. Most outfielders rely on the gaze heuristic without being able to explain how they catch a ball. Their behavior is intuitive, not consciously deliberative (Gigerenzer, 2007). In general, heuristics may be learned consciously, by instruction, or unconsciously, by trial and error learning or imitation. The process is the same, a fact overlooked by dual-process theories that align heuristics with unconsciousness and, moreover, assume different processes (see Kruglanski and Gigerenzer, 2011).

### Exaptation

The gaze heuristic was not invented by baseball outfielders. Bats, birds, fish, and other animals rely on it for intercepting prey and mates (e.g., Collett and Land, 1975). The observation that different species rely on the same heuristic invites two possible explanations, *homology* and *analogy*. Homology means that common structures between different species – here, common heuristics, – are due to a common evolutionary ancestor. Analogy means that there is a functional similarity based on something other than common ancestors. Whatever the correct explanation is, we can safely assume that the gaze heuristic evolved for predatory-prey interaction and not for baseball or cricket.

Sperber (1994) distinguished the proper domain of a cognitive module from its actual domain, that is, the domain for which a module actually evolved from a domain to which it was extended or transferred. Similarly, the term *exaptation* means that a trait or feature acquires a new function beyond its original one derived by evolution. It was introduced by Gould and Vrba (1982) as an alternative to the concept of *preadaptation* in order to emphasize that the original function was not connected to the new function. A classical example is the argument that feathers were not evolved for flight in birds, but originally had the function of temperature regulation in their ancestors, reptiles. Eventually, feathers became enlisted for a new function, sailing and, eventually, flying. I have not yet seen a discussion of exaptation with respect to heuristics, embodied or not. Here, I use the term *exaptation* in a more general sense, beyond its original biological meaning, namely, for cultural exaptation where humans find new functions for evolved heuristics. The gaze heuristic is a candidate in point. Its proper domain, or original function, is described in the next section.

## Predator-Prey Coordination

**Figure 3:**
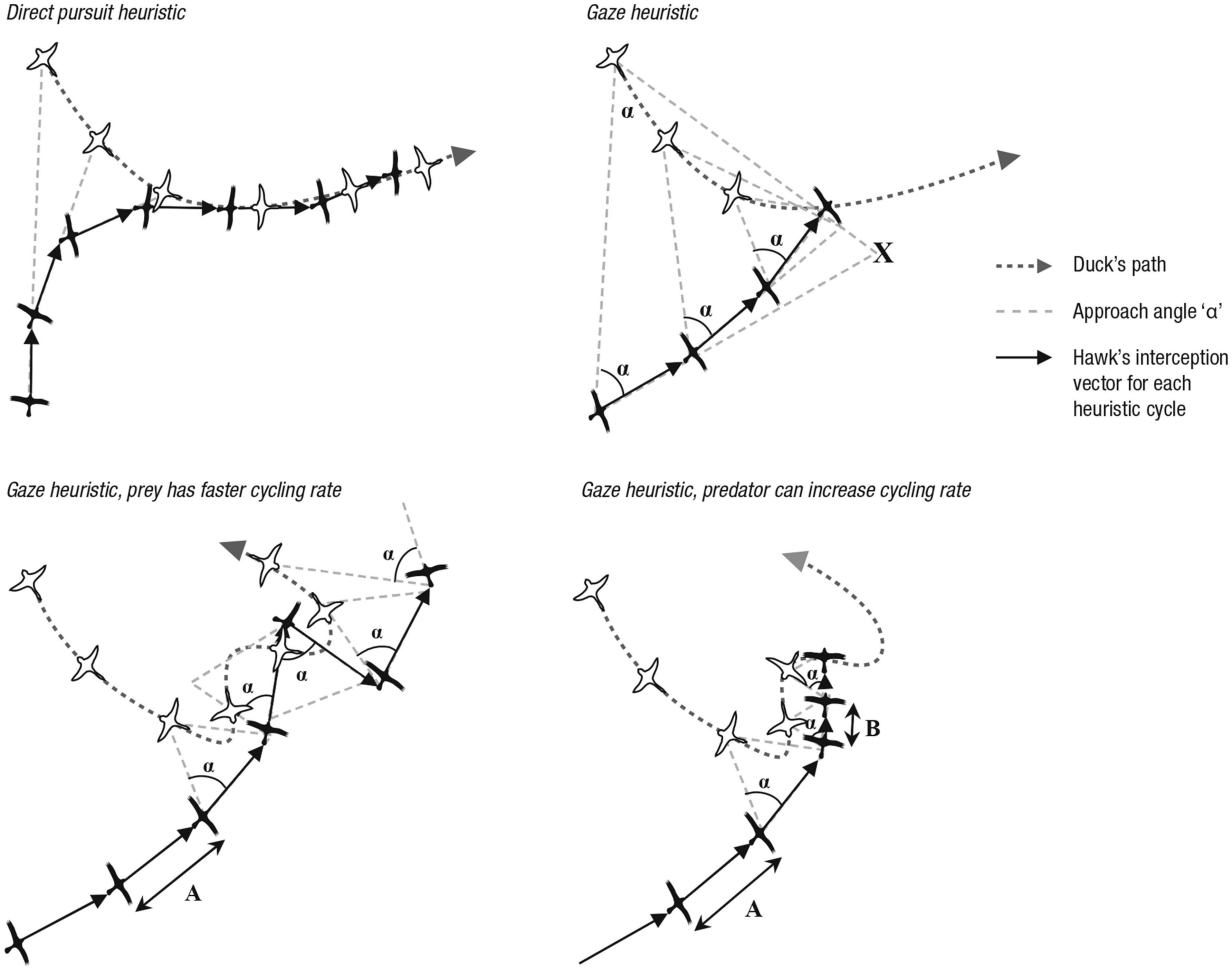
Predators (dark hawks) pursuing prey (white ducks). From top left to bottom right: *Direct pursuit heuristic:* A predator flies in the direction that is the shortest path to the prey, and adjusts the direction when the prey changes its course. If predator and prey fly at the same speed, the result is a characteristic wavering pursuit pattern. *Gaze heuristic:* A predator determines the angle α between the direct line to the prey and the initial estimate *X* of the intersection point, and then adjusts its direction to the subsequent flight path of the duck so that α remains constant. Even when both predator and prey fly at the same speed, the predator can intercept the prey. *Predator relies on gaze heuristic, but prey has faster cycling rate*: The number of adjustments an animal can make per second is its cycling rate, represented by the length *A* of its path before it can change its direction. Here, the prey has a faster cycling rate than the predator, which enables it to evade the predator. *Predator relies on gaze heuristic and can increase cycling rate*: Here, the predator has the ability to increase the cycling rate from *A* to *B*, which is higher than that of the prey, resulting in fast interception. Adapted from Hamlin (2017).

How does a hawk intercept a duck? Figure 3 (top) shows two strategies for interception. The first is *direct pursuit*, where the hawk flies straight at the duck, that is, takes the shortest path. When the duck changes its position, the hawk changes its direction accordingly, so that the distance between it and the duck is always the shortest possible. The top left panel shows a case of direct pursuit that ends in a failed interception with a characteristic wavering tail chase (Hamlin, 2017). The second strategy is a version of the gaze heuristic, where the hawk does not fly in a straight line toward the duck. Rather, it initially flies toward an expected point *X* where it would intercept the duck if the latter did not change course (top right panel). The angle α between the duck, the hawk, and the interception point *X* defines the angle of gaze. When the duck changes course, the hawk also changes its course so that the angle of gaze remains constant. In geometric terms, the angle of gaze is the base angle of a triangle with equal sides and apex *X*.

Which of the two heuristics do hawks employ? Studies with headcams mounted on hawks showed that they rely on the gaze heuristic (Kane et al., 2015). The comparison between direct pursuit and the gaze heuristic in Figure 3 indicates why: Relying on the latter allows for faster interception and avoids the wavering tail chase. Moreover, because the hawk does not fly directly toward the duck, its attack is less obvious. Only when the target is stationary do hawks rely on direct pursuit, that is, fly directly toward the prey.

To be successful in pursuit, an organism needs the ability to adjust speed and direction quickly when the angle changes (due to wind in the case of the fly ball, or due to evasive movements in the case of the duck). The number of possible adjustments per second is the *visual cycle rate.* Raptors have a visual cycling rate of about 200 per second, whereas humans have a much lower rate of about 10 per second (Hamlin, 2017). The cycling rate corresponds to the length of the path *A* before it can be adjusted to maintain a constant angle of gaze. The smaller *A* is, the faster the hawk’s cycling rate. Figure 3 (bottom left panel) shows a prey with a faster cycling rate than the hawk that avoids interception by changing its course before the hawk is able to do so. Thanks to a faster cycling rate, the prey can even get behind the predator. Although the hawk keeps the optical angle constant, it is too slow to adjust. Finally, the bottom right panel shows a successful predator that increases its cycling rate in the final stage of the pursuit from *A* to *B*.

### From Gaze to Echolocation and Whiskers

Although the gaze heuristic is named after the visual sense, it has been adapted to other senses, too. Bats rely on the equivalent of the gaze heuristic when hunting moths in darkness, but their interception is based on sound, not vision. They use an echolocation system that emits sound as a series of short “clicks” or “calls” (Denny, 2004). When a target is located, the clicks occur more frequently as the bat closes in on a prey. The echolocation version of the gaze heuristic works as described in Figure 3, except that the angle α is based on echolocation rather than visual location. In response to bats, moths have evolved bat-detecting ears capable of hearing the clicks (Hofstede and Ratcliffe, 2016). Outside the bat’s detection range, a moth’s first reaction is to fly away from the bat. If the frequency of clicks increases, meaning that the bat has detected its prey, this triggers spasms in the moth’s wings, resulting in unpredictable flight. Finally, if the clicks peak in a buzz of about 200 clicks a second, the moth’s reflex is to instantly freeze to fall out of the bat’s path. All this happens within seconds. The bat’s clicks correspond to the visual cycles of humans and hawks.

At the final stage of pursuit, the gaze heuristic is supported by tactile senses. Mammals such as cats, rats, and seals use their whiskers to locate the prey. Whiskers are an array of long, coarse hairs around the head and mouth that provide information about the prey’s position in the final milliseconds before impact (Grant et al., 2009). Experiments showed that rats were less successful in completing an interception of a mouse when their whiskers were removed, and if they did succeed, the final clean bite to the neck took longer and was messier (Hamlin, 2017).

## The Royal Air Force Discovers the Gaze Heuristic

According to a historical analysis, the Royal Air Force (RAF), after some trial and error, was the first to have discovered the gaze heuristic around the beginning of World War II (Hamlin, 2017). The problem was that the British controllers who used radar to direct fighters to enemy planes had failed to reach the required 90% interception rate. Special calculating devices and increasingly complex mathematics were introduced to crunch the numbers, but to no avail. In this situation, an impatient RAF commander demonstrated that he could do a better job by eye, meeting the 90% rate. His system was fleshed out by the Chairman of the “Committee for the Scientific Survey of Air Defence”, Sir Henry Tizard, into a fixed angle approach and taught to the controllers. This system became known as the “Tizzy Angle” and used for the remainder of the war.

**Figure 4:**
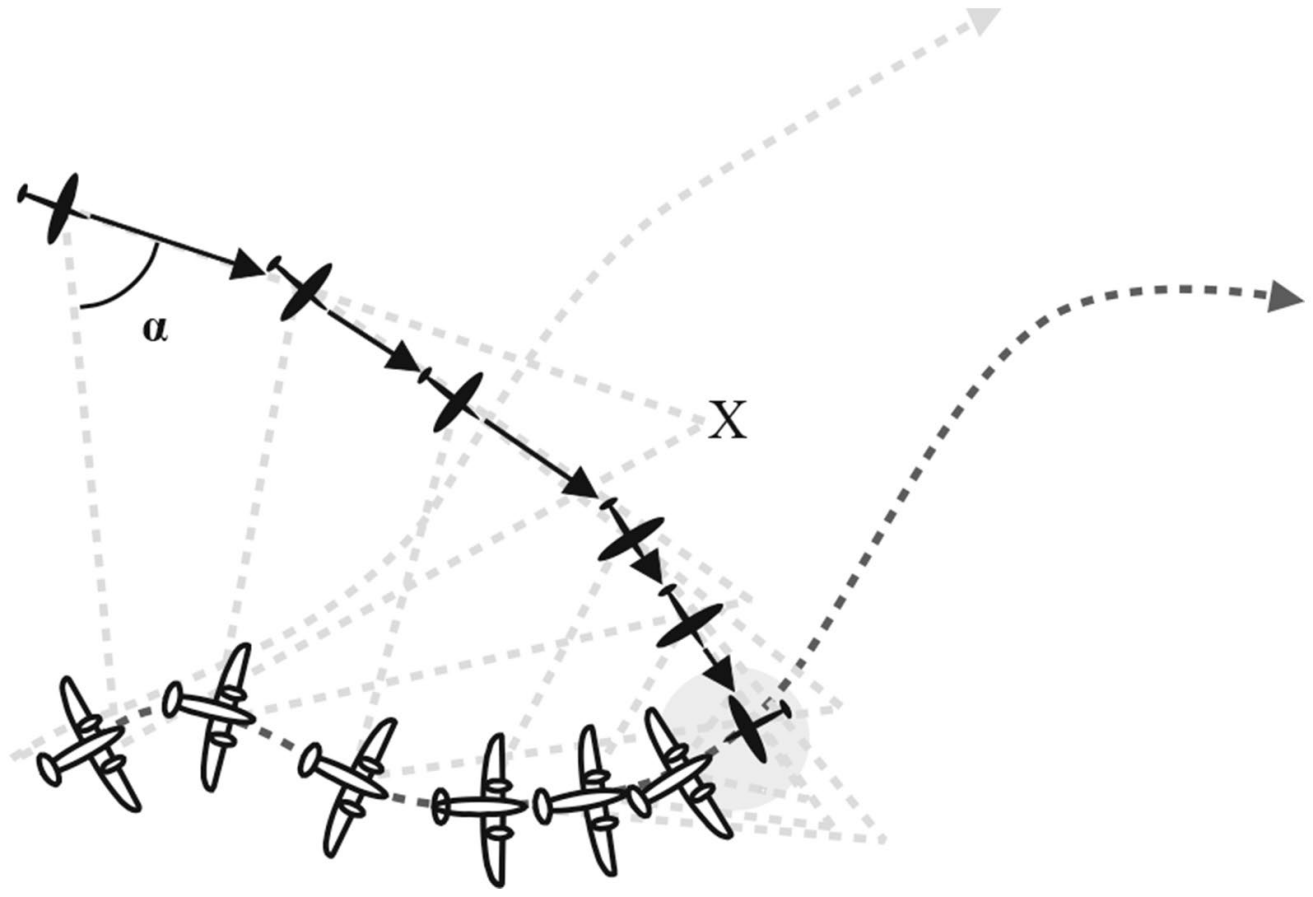
British controllers relied on the gaze heuristic to direct fighter planes to intercept German bombers. From Hamlin (2017).

After being trained to use the gaze heuristic, the British controllers no longer sent pilots directly *via* the shortest distance toward the opponent (the direct pursuit heuristic) but instead estimated an intersection point *X*, which determined the constant angle. If the bomber changed course after having recognized the fighter, the fighter was directed to change course too, but keep the angle constant. Shortly before interception, the faster fighter could turn around and meet the bomber frontally, where it was most vulnerable (Figure 4).

According to historical records and training materials, the controllers of the German Luftwaffe relied instead on a direct pursuit strategy and appear to have never discovered the gaze heuristic during World War II. In the pursuit control technique, the controller instructs the pilot (who cannot yet see the enemy plane) to fly directly toward the opponent. If the opponent changes course, the pilot is directed to also change course and take the shortest path toward the opponent. The pursuit strategy vectors the fighter behind its opponent, just as the hawk trails behind the duck in Figure 3 (top left panel), and leads to a smaller rate of interception. Although the Germans’ radar system was superior to that of the RAF in several respects, the British use of the gaze heuristic was devastating to the Luftwaffe and decisive to the Battle of Britain. Hamlin (2017) argues that the Germans might have won this battle if they had linked their high-tech radar system with a gaze-based heuristic control system. By the end of the war, the Germans were leading in missile technology, including anti-aircraft missiles based on the direct pursuit strategy, but had missed a smart heuristic.

After World War II, the United States army combined German missile technology with the British gaze heuristic system into a most successful autonomous guided weapon: the Sidewinder A1M9 short-range air-to-air missile (Hamlin, 2017). The missile is a simple, robust interception system whose “gaze” is directed at a point source of heat, which is the target. Once the missile is on its way, it makes continuous inquiries (with a rapid cycle rate) about the changes of the target’s position and adjusts its direction so that the angle of “gaze” remains constant. The Sidewinder is still in use in many nations, and new developments appear to be based on the same heuristic maintaining a constant angle of approach.

## A Research Program on Embodied Heuristics

The case study on the gaze heuristic can provide a template for a general research program on embodied heuristics. Specifically, that program addresses three core questions (see Figure 1):

*The Repertoire of Heuristics in the Adaptive Toolbox.* What are the embodied heuristics used by individuals or groups to solve problems? What sensory and motor abilities do these heuristics exploit to find efficient solutions?

*The Structure of the Environment.* What is the structure of an environment to which a given heuristics is adapted?

*Ecological rationality:* Which heuristics are likely to achieve a given goal in a given environment?

This program contrasts with a majority of theories in the cognitive sciences in two respects:

1. The body (e.g., sensory and motor abilities) and the environment “select” the heuristics and are crucial to explaining behavior. This differs from “internalist” theories that explain behavior solely by computational processes inside the mind, such as expected utility maximization, Bayesian probability updating, logical symbol manipulation, System 1/System 2 theories, and theory of mind.
2. As a consequence, behavior can often be explained by simple heuristics rather than by complex computations. An embodied heuristic can exploit innate or learned capabilities and thereby be both simple *and* accurate.
  Only the first of these two points is common to all views on embodied cognition. Although the term *embodied heuristics* is used occasionally in the literature on embodied cognition (e.g., Gallagher and Hutto, 2008, p. 27), no programs in existence develop models of embodied heuristics that can be explicated in the form of algorithms and then simulated and tested (see Table 1).

## Why Study Embodied Heuristics?

In this article, I introduced the concept of embodied heuristics and provided a case study on a particularly interesting example, the gaze heuristic. This amazing feat of evolution, a dynamic adaptive heuristic, enables animals and humans to make rapid decisions with the help of a highly automatized system superior to conscious reasoning. I end with some general insights this case study provides.

### Embodied Heuristics Are Efficient Because They Exploit Sensory and Motor Abilities

To execute an embodied heuristic requires specific sensorimotor abilities. For instance, the gaze heuristic is of little value to a robot that cannot keep its eye on a moving object against a noisy background or cannot run. In the vocabulary of AI, the software needs the proper hardware. This basic insight contrasts with most theories in decision-making that rely exclusively on logic or probability.

### Complex Problems Do Not Generally Need Complex Solutions

From machine learning to cognitive sciences, a common assumption is that the more complex a model is, the better it must perform. That is true in situations of risk or well-defined games such as chess and Go, but not in situations of uncertainty, as in interactions with humans and other animals (Katsikopoulos et al., 2020). For instance, between 2007 and 2015, Google Flu Trends tried to predict the proportion of flu-related doctor visits, based on an analysis of 50 million search terms using thousands of big data models. When predictions failed, Google engineers made the algorithm more complex instead of simpler, without any improvement. In contrast, a simple heuristic that relies on a single data point, the most recent number of flu-related doctor visits, predicts better than Google’s big data models (Katsikopoulos et al., in press). Similarly, in social encounters, heuristics based on imitation or tit-for-tat can hardly be beaten, even in well-defined games (Duersch et al., 2012). The general methodological lesson is to always test complex models against simple heuristics.

### One and the Same Heuristic Can Solve Problems in Stationary and in Nonstationary Worlds

Some scholars have hypothesized that the success of simple heuristics is restricted to stable or nonsocial worlds, and that social interactions need complex strategies (for a discussion, see Hertwig and Hoffrage, 2013). The gaze heuristic is a clear counterexample, as are tit-for-tat and heuristics relying on imitation. Moreover, in the present case, nonstationary problems of predator-prey interaction are the proper domain of the gaze heuristic; those involving inanimate objects such as fly balls are later extensions. In general, complex models with many free parameters are likely to succeed in stable, stationary worlds, while simple heuristics, like human intelligence, evolved for dealing with an uncertain, social world (Katsikopoulos et al., 2020).

### Cognition is More Than Symbol Manipulation

Research on embodied heuristics follows and extends Simon (1956) program of bounded rationality. At the same time, it contrasts with Newell and Simon’s (1972) *physical symbol hypothesis*, which assumes that symbol manipulation, as in computers, is the essence of all rational systems, implying that sensorimotor abilities are of little relevance (see Gallese et al., 2020). Cognitive and social psychologists have largely taken their inspiration from the symbol manipulation view, assuming that cognition is mainly what statistics and computer do (Gigerenzer, 1991; Gigerenzer and Goldstein, 1996). In these theories, which are often highly complex and “as-if,” neither heuristics nor their anchoring in the body play a role.

The gaze heuristic is a simple iterative heuristic that adapts to changes in flight path due to wind in case of a fly ball or due to evasion attempts in the case of prey. It can solve problems in stationary and nonstationary environments and is embodied in the sense that it requires specific sensory and motor capabilities to function efficiently. The astonishing feat is that the heuristic has enlisted different sensory capacities in different species, including vision and echolocation. It also has enlisted various motor abilities. When dogs catch a Frisbee, they implement the gaze heuristic by running (Shaffer et al., 2004); when teleost fish pursue prey, they implement the heuristic by swimming; and when hawks go after prey, they implement it by flying. Humans implement the heuristic both in two-dimensional space, such as when trying to avoid a collision with another sailboat, or in three-dimensional space, as when trying to avoid a collision in the air.

The heuristic has also inspired rethinking financial regulation. Andrew Haldane, the Bank of England’s chief economist, presented his acclaimed Jackson Hole talk entitled “The Dog and the Frisbee” on the gaze heuristic as a model for a safer world of banking. He argued for introducing simple and robust control systems in place of complex regulatory systems, which neither foresaw nor prevented the crisis of 2008 (Haldane and Madouros, 2012). Haldane used the heuristic as an analogy for robustness, not embodiment. For instance, capital requirements are estimated by calculating the value-at-risk of a bank, which may involve estimating thousands of risk factors and millions of covariation coefficients. The limited success of these estimations recalls the calculations made by the RAF before it discovered the gaze heuristic (Gigerenzer and Gray, 2017). The banking system is a fast-changing, nonstationary environment where simple rules can lead to better and more transparent decisions. The standard approach in cognitive science, however, has resembled bank regulation, based on the assumption that more complexity is always better. Journals are filled with highly parameterized models that integrate all possibly relevant information, Bayesian or otherwise. Complexity pays for well-defined situations such as games, but leads to overfitting in ill-defined situations of uncertainty.

Evolution has given us the gaze heuristic, and with it a pointer to study the ingenious solutions it has found for a brain the size of two fists. To do so, we need to embark on a systematic study of embodied heuristics in the real world.

## Data Availability Statement

The original contributions presented in the study are included in the article/supplementary material, further inquiries can be directed to the corresponding author.

## Author Contributions

The author confirms being the sole contributor of this work and has approved it for publication.

## Conflict of Interest

The author declares that the research was conducted in the absence of any commercial or financial relationships that could be construed as a potential conflict of interest.

## Publisher’s Note

All claims expressed in this article are solely those of the authors and do not necessarily represent those of their affiliated organizations, or those of the publisher, the editors and the reviewers. Any product that may be evaluated in this article, or claim that may be made by its manufacturer, is not guaranteed or endorsed by the publisher.
